# Transarterial Chemoembolization in Combination with Local Therapies for Hepatocellular Carcinoma: A Meta-Analysis

**DOI:** 10.1371/journal.pone.0068453

**Published:** 2013-07-03

**Authors:** Mingheng Liao, Jiwei Huang, Tao Zhang, Hong Wu

**Affiliations:** Department of Hepato-Biliary-Pancreatic Surgery, West China Hospital, Sichuan University, Chengdu, Sichuan, China; University of North Carolina School of Medicine, United States of America

## Abstract

**Background:**

In previous randomized trials, transarterial chemoembolization (TACE) has shown an improvement of survival rate in hepatocellular carcinoma (HCC) when combined with radiofrequency ablation (RFA), percutaneous ethanol injection (PEI) or other therapies. The aim of this meta-analysis was to evaluate the effectiveness of combination therapy of TACE with RFA, PEI, radiotherapy (RT), three-dimensional conformal radiation therapy (3D-CRT) or High-Intensity Focused Ultrasound (HIFU).

**Methods:**

Randomized or nonrandomized studies comparing TACE combined with RFA, PEI, RT, 3D-CRT or HIFU with TACE alone for HCC were included. Meta-analysis was performed using a fix-effects model in RCTs and a random-effects model among the observational studies.

**Results:**

10 randomized trials and 18 observational studies matched the selection criteria, including 2497 patients (682 in RCTs, 1815 in non-RCTs). Meta-analysis of RCTs showed that the combination of TACE and PEI ((RR)_1__-_year=1.10, 95%CI=0.99-1.22, p=0.073; (RR)_3__-_year=2.32, 95%CI=1.52-3.53, p<0.001), TACE+RT ((RR)_1__-_year=1.37, 95%CI=1.11-1.70, p=0.004; (RR)_3__-_year=2.32, 95%CI=1.44-3.75, p=0.001) were associated with higher survival rates. The results of observational studies were in good consistency with that of RCTs. Furthermore, TACE plus 3D-CRT ((RR)_1__-_year=1.22, 95%CI=1.06-1.41, p=0.005; (RR)_3__-_year=2.05, 95%CI=1.48-2.84, p<0.001) and TACE plus HIFU ((RR)_1__-_year=1.16, 95%CI=1.01-1.33, p=0.033; (RR)_3__-_year=1.66, 95%CI=1.12-2.45, p=0.011) have introduced marked survival benefit when pooling results from observational studies.

**Conclusions:**

This meta-analysis demonstrated that TACE combined with local treatments, especially PEI, HIFU or 3D-CRT could improve the overall survival status than performing TACE alone. Importantly, these results need to be validated in further high-quality clinical trials.

## Introduction

Liver cancer is the sixth most common cancer worldwide, and the incidence is still increasing. Surgical resection with complete tumor removal might be favorable in noncirrhotic patients with hepatocellular carcinoma (HCC), while transarterial chemoembolization (TACE) represents an effective treatment option for unresectable, intermediate stage HCC[1]. While the magnitude of the benefit was relatively small, TACE introduces tumor necrosis which could maintain a local tumor control, prevent tumor progression and improve survival[2].

In the 1990s, Kato has observed that the outcome of a new therapy containing both TACE and percutaneous ethanol injection (PEI) was better than performing TACE or PEI alone [3], revealing that the combination of TACE and other local therapies may have several theoretical advantages. Thus, more clinical trials were conducted to evaluate the effectiveness of TACE added another local treatment, such as radiofrequency ablation (RFA), radiotherapy (RT), three-dimensional conformal radiation therapy (3D-CRT) or high-intensity focused ultrasound (HIFU). However, the outcomes of the previous clinical trials were still confounding.

Therefore, this meta-analysis was aimed to investigate whether a combination of local therapies and TACE could improve the overall survival of HCC patients.

## Materials and Methods

The process of the meta-analysis was performed according to the Cochrane Collaboration recommendations[4]. The analysis results were reported according to the PRISMA (Preferred Reporting Items for Systematic Reviews and Meta-Analyses) statement[5].

### Search Strategy

The database of MEDLINE, EMBASE, the Cochrane Library, and Chinese BioMedical Literature Database (CBM) were searched with the following medical subject headings (MeSH): “hepatocellular carcinoma”, “chemoembolization”, “clinical trials”. All the above MeSH terms were exploded. Free text words were searched combined with additional keywords: “liver tumor”, “liver cell carcinoma”, “transarterial chemo-embolization”, “retrospective studies”. No language limitation or other restrictions such as research design was imposed. The search included literature published until December 2012 with no lower date limit. The computer search was supplemented with manual searches for references of included studies.

### Selection Process and Data Abstraction

For the study selection, article titles and abstracts were reviewed first, then full-text were obtained to assess study eligibility. Each study was evaluated and classified by two independent investigators (Liao, MH and Wu, H). Any disagreement among reviewers was resolved by discussion. The article full-text were extracted to fulfill a predefined data table which contains the characteristics of patients, tumors and outcomes.)

### Including and Excluding Criteria

Studies were included in the analysis if: (1) they were randomized controlled trials, or observational studies comparing TACE combined other supplement therapy with TACE alone; (2) there were no evidence for extrahepatic metastasis before the first TACE process; (3) overall survival was assessed as an outcome measure of the effect of the treatment. If studies were duplicates, the one with complete data was included.

Studies were excluded if they received surgery or if they were published only in abstract form. Conference proceedings or abstracts were included only if they have sufficient follow-up and clarified no surgical intervention.

### Quality Assessment

The risk of bias in RCTs was assessed following Cochrane recommendations, considering random sequence generation, allocation concealment, blinding of participants and personnel, blinding of outcome assessment, incomplete outcome data and selective reporting [6]. Each category was assessed as low, unclear or high risk of bias and summarized in a table with a plus, question mark or minus, respectively.

Observational studies were assessed by the Newcastle-Ottawa Quality Assessment Scale (NOS)[7]. This score assesses studies according to the selection of patients in the exposed and the non-exposed group, comparability of the two groups, and outcome of the single studies. A study can be rated 0-9 stars based on these criteria while 6 stars or above was considered high quality in previous studies and was included in this review.

Publication bias was evaluated by funnel plots and Egger’s regression[4].

### Statistical Analyses

The meta-analysis of RCTs and observational studies were conducted separately. The studies were divided into five subgroups according to the five different combined therapies; meanwhile, separate meta-analysis was conducted within the subgroups. In all analyses, we expressed results for treatment outcomes as relative risk (RR) with 95% confidence intervals (CIs). We used the Cochrane’s Q statistic to assess heterogeneity between studies. We used a fixed-effect model for calculations of RCTs unless there was a significant heterogeneity. Among observational studies, results were obtained using a random-effects statistical model, because of the considerable clinical heterogeneity. Additionally, the underlying relationship between study features and clinical heterogeneity was intended to be revealed through a meta-regression analysis. Begg’s funnel plots and Egger’s regression asymmetry test was used to examine potential publication bias. For all analyses, P<0.05 was considered statistically significant. Statistical analyses were performed with the software programs Review Manager (Version 5.2), and Stata (Version 12.0, Stata Corp LP, College Station, TX, USA).

## Results

### Description of the studies

**Figure 1:**
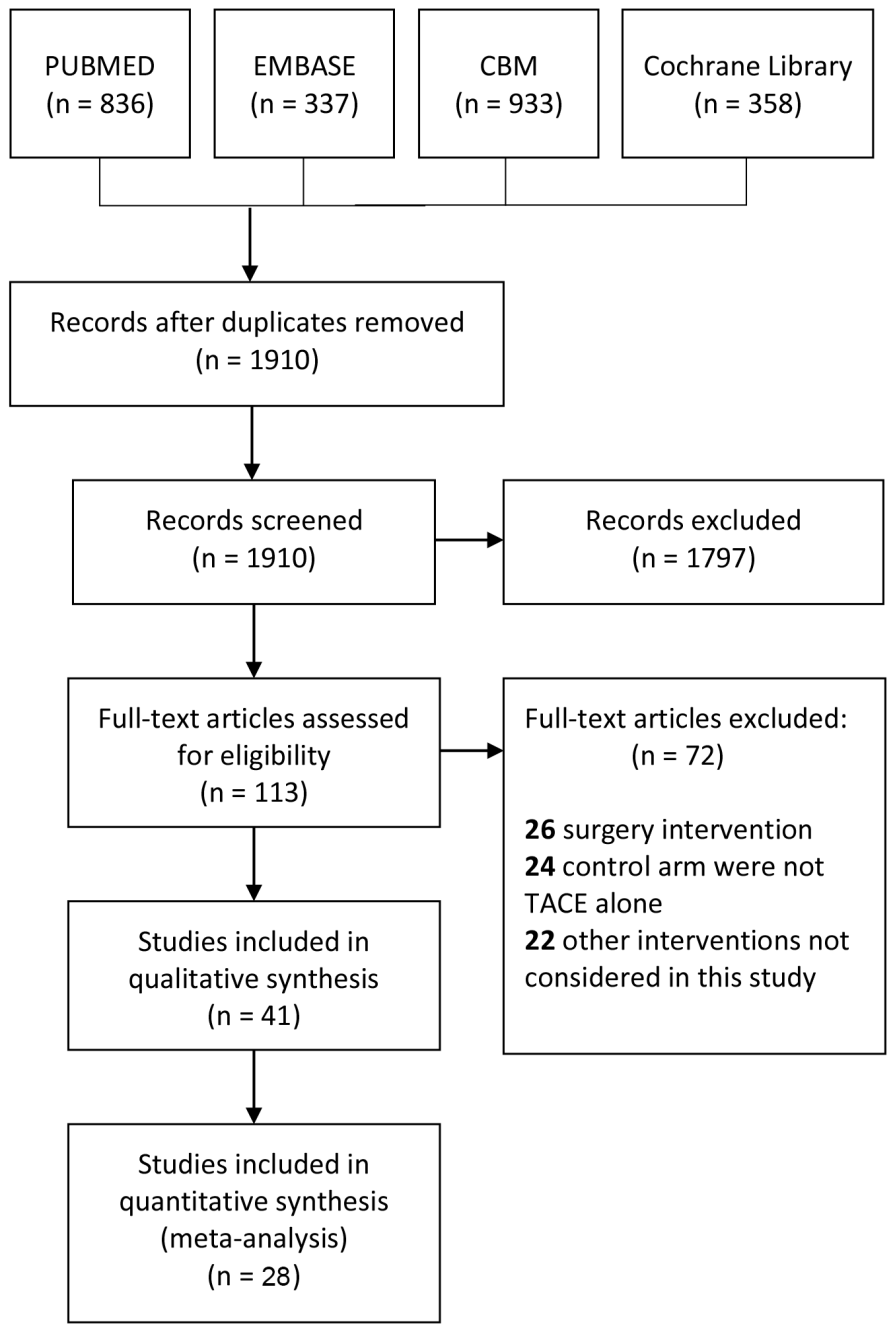
Identification of eligible studies from different databases.

The literature search yielded a total of 1910 studies. After reviewing the titles and abstracts, there were 113 studies left for the quality assessment. The full-texts had been carefully evaluated, 22 studies were excluded for involving other interventions, 26 for surgical intervention, 24 for interventions other than TACE alone in the control arm, 6 for high-risk of bias [35–40], 1 retracted after publication [41], 1 duplicate article published in local language [42] and 5 for insufficient follow-up [40,43–46]. There were 10 randomized controlled trials, 11 prospective and 7 retrospective observational studies fulfilled the inclusion criteria and were chosen to be reviewed (Figure 1). The main characteristics of randomized trials (Table 1) and observational studies (Table 2) were listed.

**Table 1:** The characteristics of randomized trials included in the meta-analysis.

Study	Arms	Patients n.	Gender (male)	Child-Pugh Class (A/B/C)	Tumor size (mean±SD, cm)	Number of tumor (1/>2)	1-year survival	3-year survival
Zhao et al. [8]	TACE+3D-CRT	49	32	49/0/0	all<6	N.R.	82%	43%
	TACE	47	28	47/0/0			55%	15%
Liu et al. [9]	TACE+HIFU	43	67.9%	45/33/0	all > 5	40/38	74.4%	16.3%
	TACE	35					48.6%	0%
Bartolozzi et al. [10]	TACE+PEI	26	19	14/12/0	4.84±1.44	18/8	100%	72.2%
	TACE	27	22	11/16/0	5.09±1.36	14/13	92.6%	43.4%
Beckeret al. [11]	TACE+PEI	27	20	17/10/0	n=17 > 5cm	13/14	61.5%	N.R.
	TACE	25	21	22/3/0	n=17 > 5cm	9/16	62.9%	N.R.
Xu et al. [12]	TACE+PEI	23	N.R.	23/0/0	all > 5cm	23/0	88%	21%
	TACE	22		22/0/0		22/0	59%	0%
Yamamoto et al. [13]	TACE+PEI	50	42	17/23/10	N.R.	22/28	95%	50%
	TACE	50	45	20/19/11		26/24	92.5%	20%
Yang et al. [14]	TACE+RFA	24	18	11/5/1	6.6±0.6	5/19	68.3%	N.R.
	TACE	11	8	10/5/0	6.4±1.0	7/4	53.2%	
Leng et al. [15]	TACE+RT	36	27	N.R.	9.7	34/2	74.8%	40.4%
	TACE	39	27		10.4	34/5	61.3%	19.8%
Wang et al. [16]	TACE+RT	20	18	N.R.	N.R.	N.R.	50%	N.R.
	TACE	20	19				33.3%	
Wang et al. [17]	TACE+RT	54	43	N. R.	n=19 > 5cm	49/5	76.5%	42.1%
	TACE	54	44		n=22 > 5cm	50/4	53.2%	18.6%

N.R., not reported

**Table 2:** The characteristics of observational studies included in the meta-analysis*.

Study	NOS	Arms	Patients n.	Child-Pugh Class (A/B/C)	Tumor size (mean±SD, cm)	Portal vein thrombus	1-year survival	3-year survival
Lan et al.[18]	******	TACE+3D-CRT	42	N.R.	All > 3cm	12	57.1%	26.2%
		TACE	60			14	61.7%	16.7%
Li et al.[19]	******	TACE+3D-CRT	41	27/14/0	All > 3cm	N.R.	73,2%	41.9%
	*	TACE	41	23/18/0			54.8%	12.8%
Liu et al.[20]	******	TACE+3D-CRT	54	40/14/0	n=15 > 10cm	10	66.5%	37.4%
	*	TACE	60	43/17/0	n=16 > 10cm	11	53.9%	17.8%
Shang et al.[21]	******	TACE+3D-CRT	40	N.R.	All<6cm	N.R.	78%	34%
		TACE	36				50%	18%
Zeng et al.[22]	******	TACE+3D-CRT	54	44/10/0	n=44 > 5cm	N.R.	71.5%	24%
		TACE	149	114/35/0	n=128 > 5cm		59.6%	11.1%
Li et al.[23]	******	TACE+HIFU	38	34/4/0	9.3±2.2	N.R.	71.1%	N.R.
		TACE	30	27/3/0			46.7%	
Peng et al.[24]	******	TACE+HIFU	20	N.R.	n=14 > 10cm	N.R.	65%	N.R.
		TACE	32		n=21 > 10cm		62.5%	
Ye et al.[25]	******	TACE+HIFU	56	15/35/6	n=37 > 5cm	9	82.3%	39.2%
		TACE	50	14/31/5	n=39 > 5cm	8	68%	21.3%
Zhang et al.[26]	******	TACE+HIFU	55	29/48/28	Mean 4.5cm	N.R.	80%	47.3%
		TACE	50				74%	30%
Greten et al.[27]	******	TACE+PEI	52	N.R.	N.R.	N.R.	92%	12.2%
		TACE	49				54%	33.6%
Kamada et al.[28]	******	TACE+PEI	32	11/21/0	2.2±0.5	N.R.	90%	65%
	**	TACE	37	10/27/0	2.4±0.6		86%	44%
Kato et al.[3]	******	TACE+PEI	24	19/5/0	6.52	N.R.	87%	39.7%
	*	TACE	22	17/5/0	7.09		50.7%	8.5%
Lubienski et al.[29]	******	TACE+PEI	22	10/8/4	7.1±3.3	N.R.	55%	22%
	*	TACE	28	16/8/4	8.6±4.5		21%	4%
Qu et al.[30]	******	TACE+PEI	142	53/83/6	9.6	76	62%	N.R.
	*	TACE	170	63/107/0	9.0	89	34.1%	
Cheng et al.[31]	******	TACE+RT	17	17/0/0	8.6±4.1	2	82.3%	N.R.
	*	TACE	16	16/0/0	5.4±4.5	3	68.7%	
Guo et al.[32]	******	TACE+RT	76	63/13/0	All > 5cm	14	64%	28.6%
	**	TACE	89	74/15/0	All > 5cm	22	39.9%	9.5%
Shim et al.[33]	******	TACE+RT	38	33/5/0	10.2	12	65.8%	N.R.
		TACE	35	32/3/0	9.5	10	32.3%	
Song et al.[34]	******	TACE+RT	28	11/17/0	9.2±3.6	8	72.4%	39.6%
	*	TACE	28	13/15/0	9.0±3.0	11	53.4%	20.8%

* A detailed content of Table 2 has been uploaded as the supporting information (Table S1).

N.R., not reported

Ten RCTs were published between 1995 and 2008, originated from Japan [13], Italy [10], Germany [11] and China [8,9,12,14–17]. A total of 682 patients, which had been randomized into combined and monotherapy, were included. In the treat arm, transarterial chemoembolization (TACE) was combined with radiofrequency ablation (RFA), percutaneous ethanol injection (PEI), radiotherapy (RT), three-dimensional conformal radiation therapy (3D-CRT) or high-intensity focused ultrasound (HIFU). While in the control arm, the treatment is TACE alone. Totally 1815 patients were included in eighteen prospective and retrospective observational studies. Among those, 833 patients were in the treat arm with combined therapies, compared with 982 patients who received monotherapy.

The number of patients in each control arm ranging from 22 to 170. The mean age was 42.9, the percentage of men ranged from 60.9% [3] to 93.3% [26], and the proportion of liver function below Child-Pugh A ranged from 0% [31] to 72.4% [25]. Data of the hepatic tumors were provided in different aspects. The number of tumors (solitary versus multiple) were reported in 19/28 articles, the diameter of tumors varied from <3cm [28] to >10cm [32–34], the infiltration of portal vein thrombus was mentioned in some studies, while hepatitis status was mostly described.

### Quality Assessment

**Figure 2:**
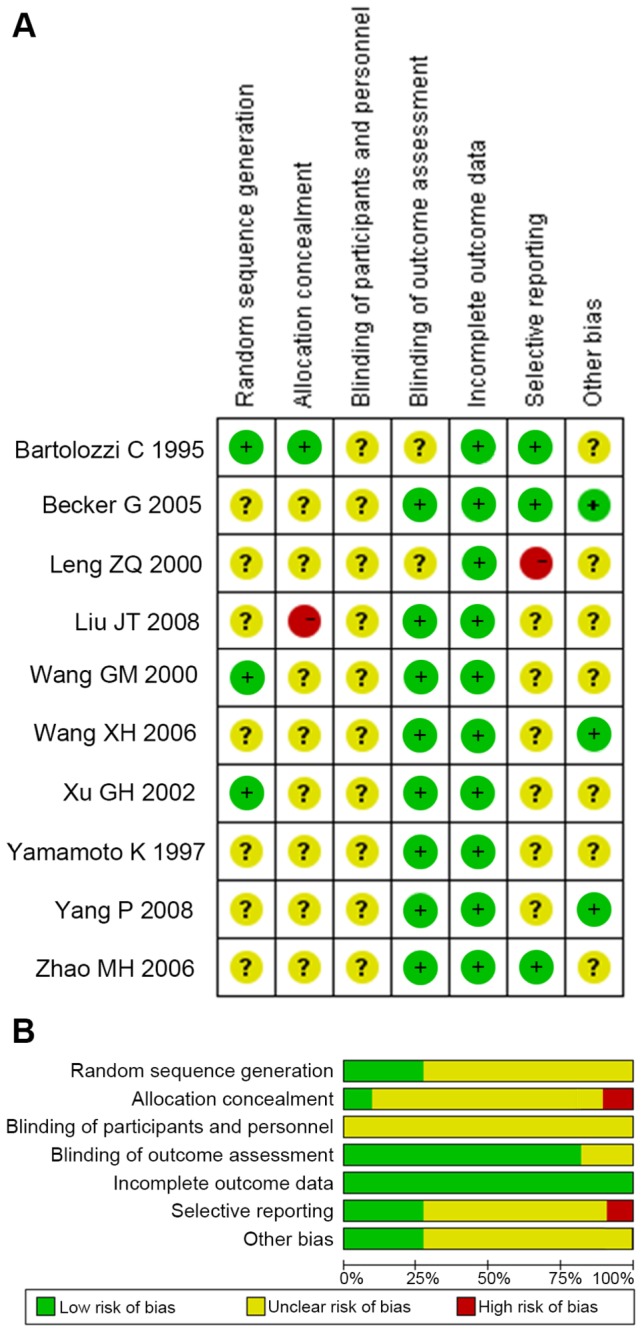
Assessment of risk of bias. **A** Summary of risk of bias for each randomized trial assessed by Cochrane Collaboration’s tool. **B** Risk of bias graph about each risk of bias item presented as percentage across all included randomized trials.

The quality assessment of RCTs were performed using Cochrane Collaboration’s tool. The most obvious risk of bias in the RCTs was the blinding procedure. However, blinding techniques were hardly feasible because of the different treatment procedure and the associated adverse effects. Meanwhile, all RCTs had no adequate description of allocation concealment, thus the risk of bias is apparent. Moreover, the tumor feature was hardly comparable, introducing an unclear risk of selection bias. Risk of attrition bias was not presented across studies. The overall risk of all types of bias in the RCTs was generally low to unclear (Figure 2).

The heterogeneity among the observational studies was considerable. The most typical methodological flaw was a risk of selection bias. The tumor number and size had significant differences between groups, and only a few studies reported the portal vein infiltration [3,18,20,30–34]. Attrition bias was also apparent for the number of patients lost in follow-up was only partly concerned. Reporting bias was not so obvious since we choose the survival rate as the outcome measure (Table 2).

### Survival rates

#### One-year survival

**Figure 3:**
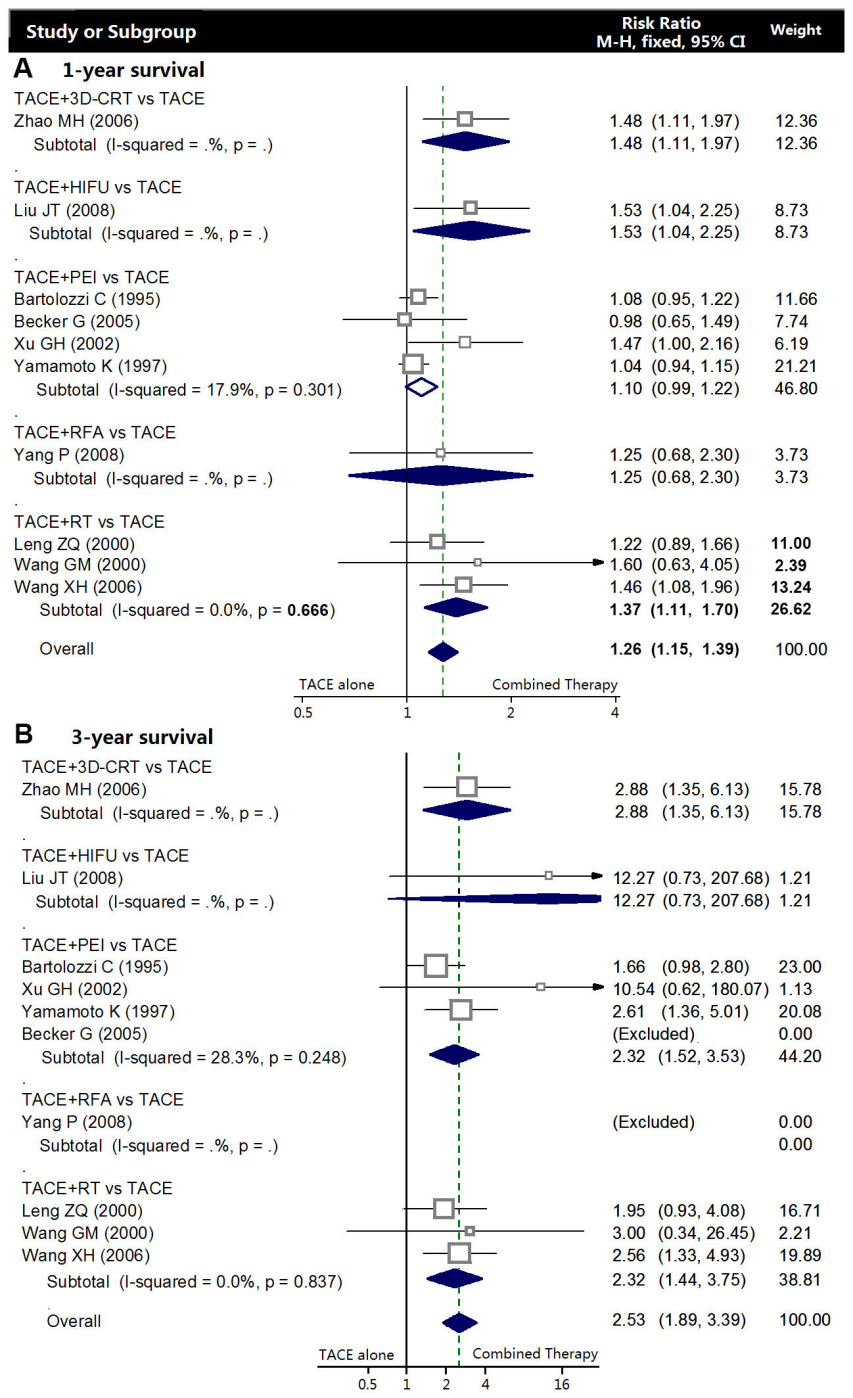
Meta-analysis of randomized clinical trials. Comparing the combined therapy with TACE alone in terms of overall survival rates. **A** Meta-analysis of one-year survival. **B** Meta-analysis of three-year survival.

**Figure 4:**
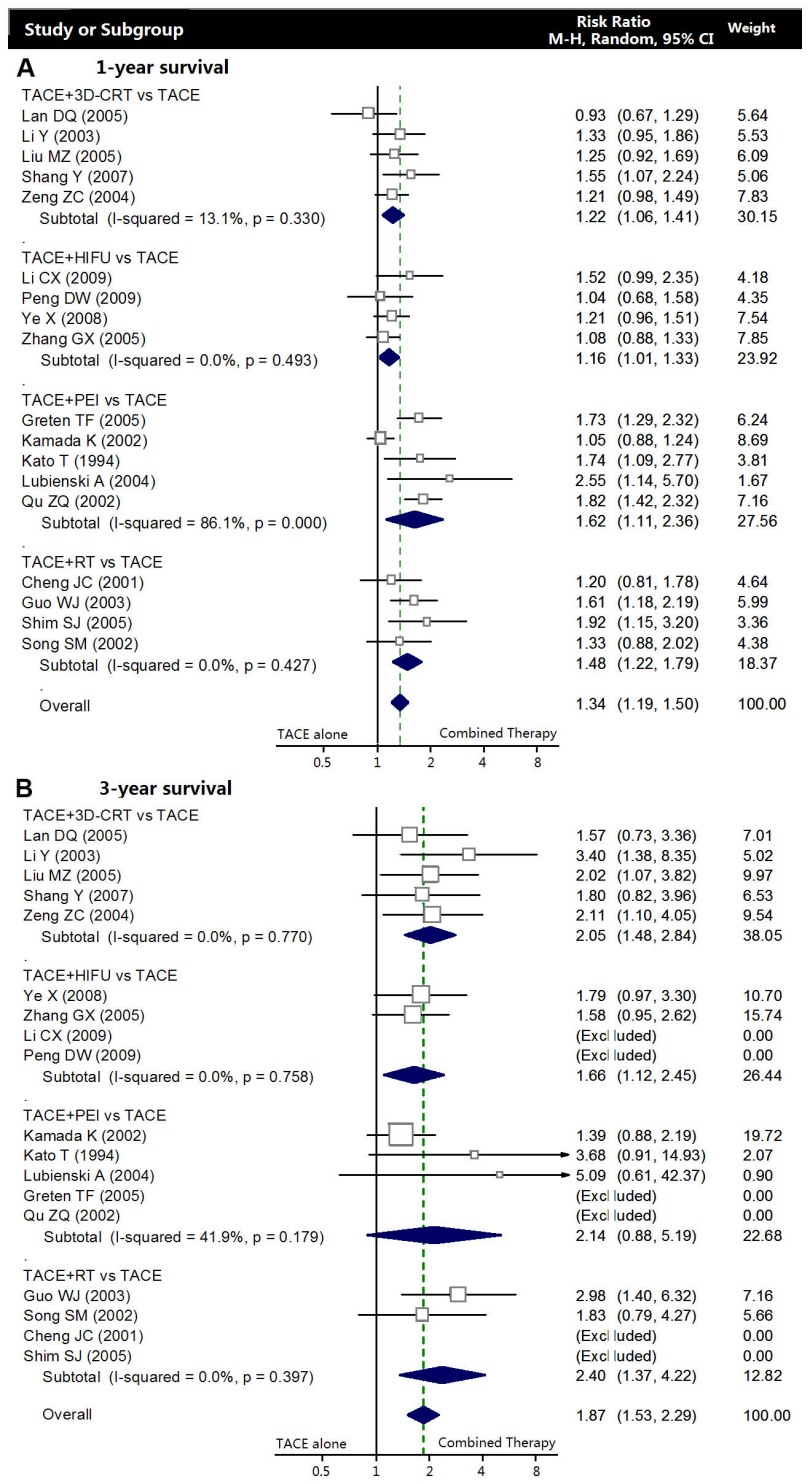
Meta-analysis of observational studies. Comparing the combined therapy with TACE alone in terms of overall survival rates. **A** Meta-analysis of one-year survival. **B** Meta-analysis of three-year survival.

In meta-analysis of randomized controlled trials, there was no marked heterogeneity in each subgroup, thus the fixed-effects model was chosen to pool the result. There was only one RCT included in TACE+RFA, TACE+HIFU, and TACE+3D-CRT subgroup. Data showed the combination of TACE+RT was associated with a higher one-year survival rate compared with TACE alone (RR: 1.37, 95% CI: 1.11-1.70; p=0.004), while the survival benefit of TACE+PEI (RR: 1.10, 95% CI: 0.99-1.22; p=0.073) was relatively small (Figure 3). A random-effect model yielded that TACE+3D-CRT (RR: 1.22, 95% CI: 1.06-1.41; p=0.005), TACE+HIFU (RR: 1.16, 95% CI: 1.01-1.33; p=0.033), and TACE+RT (RR: 1.48, 95% CI: 1.22-1.79; p<0.001) had significant survival benefits, especially the TACE plus PEI combination represented a more significant improvement of one-year survival (RR: 1.61, 95% CI: 1.12-2.33; p<0.001) (Figure 4).

Comparing the combined therapy with TACE alone in terms of overall survival rates. **A** Meta-analysis of one-year survival. **B** Meta-analysis of three-year survival.

Logistic regression analysis was taken to identify potential sources of heterogeneity among the studies. Under univariate logistic regression, higher percentage of portal vein thrombosis was found to be associated with a worse survival (P<0.05). While gender, Child-Pugh class, Hepatitis and other study characteristics showed little relationship (Table 3).

**Table 3:** Predictors of 1-year survival among all studies.

	**Outcome (1-Year Survival)**			
**Study Characteristics**	**No. of Studies**	**β**	**SE**	**P**
Publication year	28	0.011	0.010	0.289
Study location*	28	0.094	0.150	0.530
Study Design**	28	0.055	0.097	0.581
Male sex, %	28	-0.396	0.431	0.377
HBV, %	7	0.200	0.145	0.227
HCV, %	6	-0.144	0.245	0.588
Child-Pugh class A, %	21	0.329	0.169	0.066
Child-Pugh class B, %	21	-0.230	0.207	0.280
Solitary,%	17	-0.072	0.246	0.775
Portal vein thrombosis, %	11	1.117	0.285	0.004

For study location, “1” corresponds to Asia-Pacific studies; “0” to European studies.

For study design, “1” corresponds to randomized controlled trials; “2” to observational studies.

HCV, hepatitis C virus; HBV, hepatitis B virus; SE, standard error

#### Three-year survival

Data for three-year survival rate were reported in 8 RCTs and 12 observational studies. Meta-analysis of RCTs indicated that combined therapies, especially the additional PEI (RR: 2.32, 95%CI 1.52-3.53; p<0.001) and RT (RR 2.32, 95%CI=1.44-3.75; p=0.001) significantly improved the three-year survival compared with TACE alone (Figure 3). A random-effect model yielded a similar result from the observational studies, furthermore, it also showed TACE+HIFU (RR: 1.66, 95%CI 1.12-2.45; p=0.011) and 3D-CRT treatment (RR: 2.05, 95%CI 1.48-2.84; p<0.001) were associated with higher three-year survival. However, the researches of TACE+RFA/TACE were inadequate to obtain a comment (Figure 4).

### Adverse effect

Most studies reported no serious side effects. The most common adverse effects were the postembolization syndrome: fever, mild nausea and mild abdominal pain, and were usually self-limited. Mild elevation of serum aminotransferase level (ALT or AST) or total bilirubin (TB) was reported mainly in PEI intervention [11,47,48], they were transient and patients recovered in a short time. In addition, radiotherapies showed more side effects such as leukocyte count decline [8,17,21] or total bilirubin level rise [20,21,34]. Especially, the increased level of TB in TACE+RT subgroup has a statistically significance when compared with TACE alone [49]. Lastly, four studies reported the development of radiation-induced liver disease [18,31,33,39], but no difference was found between the intervention and control arms.

### Publication bias

The Begg’s and the Egger’s test for one-year and three-survival did not show any evidence of publication bias (Figure S1).

## Discussion

Transarterial chemoembolization has been used as a palliative tool in neoadjuvant therapy, in bridging therapy before transplantation, and even as an alternative to resection [50–52]. While previous meta-analysis showed that the combination of TACE and RFA, RT or PEI was associated with higher survival rates [49,53–57], on closer examination there are some potential methodological issues. The main problems may be referred to the heterogeneity of observational studies which were counted in pooling the results. Also, there was a potential risk of bias for clinical performance when different interventions were provided in the control arm. Since having addressed the above issues, several attempts were taken to improve the methodological quality and strength of evidence. Our meta-analysis was performed separately among RCTs and observational studies. Secondly, as previous studies had already proved that different chemotherapeutic drugs or embolization methods may have the similar effect [58–60], the control arm was limited to TACE only. Through the above measures, the combination therapies, including PEI, RFA, RT, 3D-CRT and HIFU, were methodologically comparable.

Our meta-analysis demonstrated that several combination therapies, mainly TACE+PEI, TACE+HIFU, TACE+RT, TACE+3D-CRT, and TACE+RFA were more beneficial than taking TACE alone among multinodular or unresectable HCC patients. Additionally, this was in consistent with previous clinical trials comparing TACE+RFA with RFA [41,61–63], TACE+PEI with PEI [45,64], or TACE+RT with RT [65]. In a word, the present studies indicate that these multimodal therapies bring more survival benefit than TACE or local therapy alone. Though observational studies observed the survival improvement in TACE+HIFU/TACE, however, the extent of improvement was rather small in the RCT. Meanwhile, the researches of TACE+RFA/TACE were inadequate to obtain a comment, thus further well-organized randomized trials were needed to confirm these findings. Moreover, meta-regression suggests that portal vein thrombus was related to a worse survival, but the impact of other factors such as gender, liver function or hepatitis was not significant in this study.

Most adverse effects were mild and transient, but radiotherapy for the treatment of HCC aroused concerning because of the irradiation of the normal liver tissue during a whole-liver radiation. Compared with traditional radiotherapy, 3D-CRT allowed us to conduct a more targeted radiation, and minimized the toxicity. These advantages facilitated the technical improvement. We found the trend that all included studies before the year 2000 chose RT, but studies after 2003 mostly adopted 3D-CRT. At last, though most included studies confirmed there was no serious adverse effect and the acute liver failure was so rare, the safety of the combined therapies was still one of the most concerned aspects and needs to be further evaluated.

This study may have several possible limitations. The Cochrane Library’s tool was used to assess the risk of bias of RCTs, suggested that there were higher risks for selection bias, performance bias or reporting bias among the included trials. The risk of bias was rather prominent in the randomization process. However, the blinding or the allocation concealment was hardly possible in the clinical practice. Moreover, the meta-analysis was performed using summary data, but the different characteristics of HCC patients were closely related to the survival and treatment outcome. A more practical comparison of survival should be achieved with a meta-analysis of individual patient data. Heterogeneity of observational studies was apparent. Though the sample size of the included studies was almost adequate, the inconsistency among these studies was not surprising. The potential biases may be referred to the selection of patients, the microvascular infiltration, tumor number and size, and different stage of liver function.

In conclusion, the combination of TACE and another local therapy including PEI, HIFU, or 3D-CRT could offer a more effective treatment for intermediate or advanced HCC patients than TACE alone. Importantly, these results need to be validated in further high-quality clinical trials.

## Supporting Information

Checklist S1 Prisma Checklist. (DOC)

Figure S1 The funnel plot for one-year and three-survival. **A** Funnel plot of one-year did not show any evidence of publication bias. **B** Funnel plot of three-year did not show any evidence of publication bias.
(TIF)

Table S1 The characteristics of observational studies included in the meta-analysis. (DOCX)
