# Supplementary figures and images for: Transarterial Chemoembolization in Combination with Local Therapies for Hepatocellular Carcinoma: A Meta-Analysis
Source: PLoS One. 2013 Jul 3;8(7):e68453. doi: 10.1371/journal.pone.0068453 (PMC3701086; doi:10.1371/journal.pone.0068453)

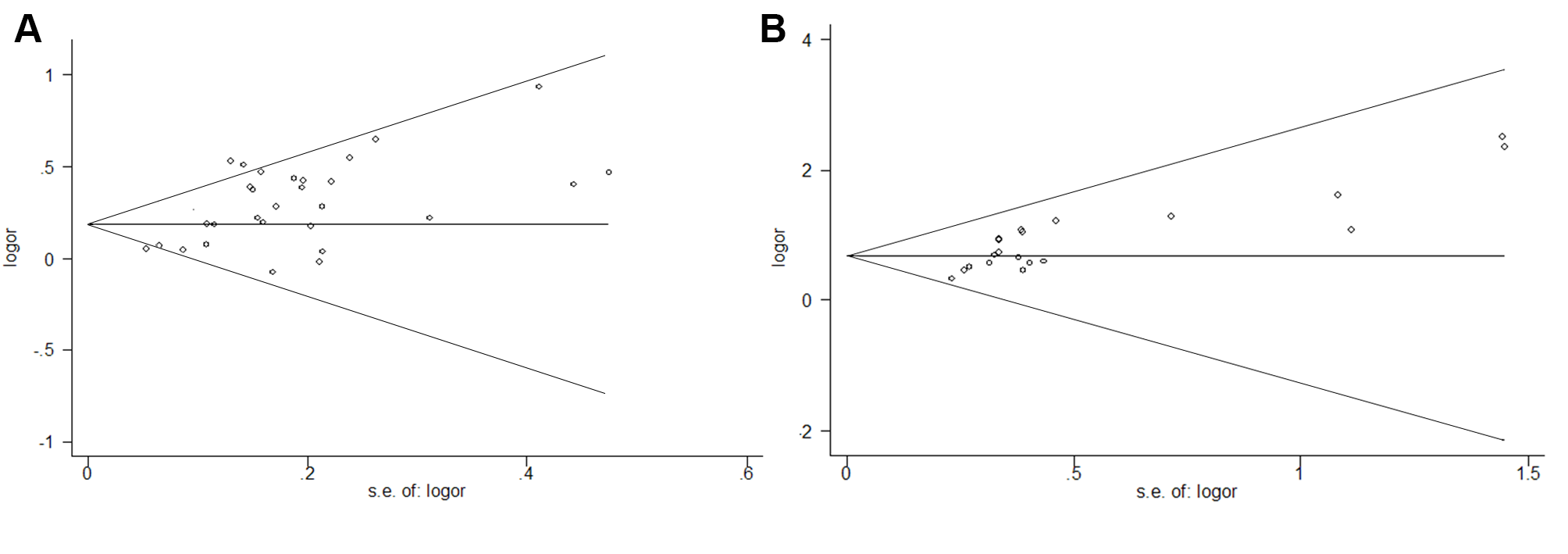

Supplement: Figure S1 — A Funnel plot of one-year did not show any evidence of publication bias. B Funnel plot of three-year did not show any evidence of publication bias. (TIF) [file pone.0068453.s002.tif]
