# Supplementary material for: Transarterial Chemoembolization in Combination with Local Therapies for Hepatocellular Carcinoma: A Meta-Analysis
Source: PLoS One. 2013 Jul 3;8(7):e68453. doi: 10.1371/journal.pone.0068453 (PMC3701086; doi:10.1371/journal.pone.0068453)
Supplement: Table S1 — (DOCX) [file pone.0068453.s003.docx]

Table S1. The characteristics of observational studies included in the meta-analysis

| Study | Design | NOS | Arms | Patients n. | Gender (male) | Child-Pugh Class(A/B/C) | Tumor size (mean±SD,cm) | Number of tumor (1/>2) | Portal vein thrombus | 1-year survival | 3-year survival |
| --- | --- | --- | --- | --- | --- | --- | --- | --- | --- | --- | --- |
| Lan et al.[18] | Prospective | ****** | TACE+3D-CRT | 42 | 78.4% | N.R. | All>3cm | 35/7 | 12 | 57.1% | 26.2% |
|  |  |  | TACE | 60 |  |  |  | 49/11 | 14 | 61.7% | 16.7% |
| Li et al.[19] | Prospective | ****** | TACE+3D-CRT | 41 | 30 | 27/14/0 | All>3cm | 32/9 | N.R. | 73,2% | 41.9% |
|  |  | * | TACE | 41 | 30 | 23/18/0 |  | 34/7 |  | 54.8% | 12.8% |
| Liu et al.[20] | Prospective | ****** | TACE+3D-CRT | 54 | 41 | 40/14/0 | n=15>10cm | 16/38 | 10 | 66.5% | 37.4% |
|  |  | * | TACE | 60 | 42 | 43/17/0 | n=16>10cm | 16/44 | 11 | 53.9% | 17.8% |
| Shang et al.[21] | Prospective | ****** | TACE+3D-CRT | 40 | 24 | N.R. | All<6cm | N.R. | N.R. | 78% | 34% |
|  |  |  | TACE | 36 | 24 |  |  |  |  | 50% | 18% |
| Zeng et al.[22] | Retrospective | ****** | TACE+3D-CRT | 54 | 50 | 44/10/0 | n=44>5cm | 37/17 | N.R. | 71.5% | 24% |
|  |  |  | TACE | 149 | 134 | 114/35/0 | n=128>5cm | 94/56 |  | 59.6% | 11.1% |
| Li et al.[23] | Retrospective | ****** | TACE+HIFU | 38 | 32 | 34/4/0 | 9.3±2.2 | 28/10 | N.R. | 71.1% | N.R. |
|  |  |  | TACE | 30 | 25 | 27/3/0 |  | 22/8 |  | 46.7% |  |
| Peng et al.[24] | Retrospective | ****** | TACE+HIFU | 20 | 17 | N.R. | n=14>10cm | 20/0 | N.R. | 65% | N.R. |
|  |  |  | TACE | 32 | 27 |  | n=21>10cm | 32/0 |  | 62.5% |  |
| Ye et al.[25] | Retrospective | ****** | TACE+HIFU | 56 | 34 | 15/35/6 | n=37>5cm | N.R. | 9 | 82.3% | 39.2% |
|  |  |  | TACE | 50 | 31 | 14/31/5 | n=39>5cm |  | 8 | 68% | 21.3% |
| Zhang et al.[26] | Retrospective | ****** | TACE+HIFU | 55 | 53 | 29/48/28 | Mean 4.5cm | N.R. | N.R. | 80% | 47.3% |
|  |  |  | TACE | 50 | 45 |  |  |  |  | 74% | 30% |
| Greten et al.[27] | Retrospective | ****** | TACE+PEI | 52 | N.R. | N.R. | N.R. | N.R. | N.R. | 92% | 12.2% |
|  |  |  | TACE | 49 |  |  |  |  |  | 54% | 33.6% |
| Kamada et al.[28] | Prospective | ****** | TACE+PEI | 32 | 25 | 11/21/0 | 2.2±0.5 | 18/14 | N.R. | 90% | 65% |
|  |  | ** | TACE | 37 | 22 | 10/27/0 | 2.4±0.6 | 21/16 |  | 86% | 44% |
| kato et al.[3] | Prospective | ****** | TACE+PEI | 24 | 15 | 19/5/0 | 6.52 | N.R. | N.R. | 87% | 39.7% |
|  |  | * | TACE | 22 | 13 | 17/5/0 | 7.09 |  |  | 50.7% | 8.5% |
| Lubienski et al.[29] | Retrospective | ****** | TACE+PEI | 22 | 18 | 10/8/4 | 7.1±3.3 | 8/14 | N.R. | 55% | 22% |
|  |  | * | TACE | 28 | 21 | 16/8/4 | 8.6±4.5 | 15/13 |  | 21% | 4% |
| Qu et al.[30] | Prospective | ****** | TACE+PEI | 142 | 82% | 53/83/6 | 9.6 | 18/124 | 76 | 62% | N.R. |
|  |  | * | TACE | 170 |  | 63/107/0 | 9.0 | 21/149 | 89 | 34.1% |  |
| Cheng et al.[31] | Prospective | ****** | TACE+RT | 17 | 15 | 17/0/0 | 8.6±4.1 | N.R. | 2 | 82.3% | N.R. |
|  |  | * | TACE | 16 | 13 | 16/0/0 | 5.4±4.5 |  | 3 | 68.7% |  |
| Guo et al.[32] | Prospective | ****** | TACE+RT | 76 | 68 | 63/13/0 | All>5cm | 51/25 | 14 | 64% | 28.6% |
|  |  | ** | TACE | 89 | 75 | 74/15/0 | All>5cm | 59/30 | 22 | 39.9% | 9.5% |
| Shim et al.[33] | Prospective | ****** | TACE+RT | 38 | 32 | 33/5/0 | 10.2 | 38/0 | 12 | 65.8% | N.R. |
|  |  |  | TACE | 35 | 23 | 32/3/0 | 9.5 | 35/0 | 10 | 32.3% |  |
| Song et al.[34] | prospective | ****** | TACE+RT | 28 | 26 | 11/17/0 | 9.2±3.6 | N.R. | 8 | 72.4% | 39.6% |
|  |  | * | TACE | 28 | 24 | 13/15/0 | 9.0±3.0 |  | 11 | 53.4% | 20.8% |

N.R., not reported
